# Mr. Pulley, on the Bony Covering of the Brain

**Published:** 1805-12

**Authors:** John Pulley

**Affiliations:** Bedford


### To the Editors of the Medical and Physical Journal.

GENTLEMEN,

I Have just read the observations of Dr. Walker, on the bony covering of the brain, and I must at once allow that they are equally ingenious and erroneous. At the first blush, there appears something very plausible in the idea, that the equal and temperate resistance of an osseous investment should so regulate the vital current, as not to impede pede a due performance of those actions so essential to our existence. But disarm this conjecture of its specious robe, and it must vanish into empty air. I hope that my expressions will not be misapprehended; I have no wish to treat with levity the opinions of a man whom I cannot but respect, but I must maintain that facts, as they stand, will go to controvert his theory.

It will be admitted by all, that the cranial bones serve to lodge and defend their contained parts; and that their figure, or formation of skull, is admirably adapted for such purposes: but this figure is not moulded for the internal parts, they fashion it themselves, and take that capacity of building which their offices may require.

Dr. Walker asks, if a bony case did not exist, under great increased action of the heart, "to keep every part of the medullary mass firm in its place, would there not be a rupture of delicate parts; interruption of their ineffably and incomprehensibly curious functions, or intestine motions? Would not intellect become immediately deranged? Would not death soon close the scene?"—I answer, No. The cranium has but to lodge and defend its contents, and its figure and capacity are determined by them. What resistance is given to the internal parts in the infant state? none: ossification is then very imperfect, and the envelope is not asked to resist, but is made to yield; yet at this time, if resistance ever were required, it would seem more peculiarly requisite, as the hurry and vigour of circulation are greater than at any future period; but if obtained, that scene of fatality must inevitably ensue, which the Doctor would so much dread from its want of application.

If the circulation of the blood through the brain does not require tempering by an osseous covering in the infant state, should it be wanted at an adult age? This office is performed by the blood-vessels, which are so distributed within the cranium, as generally to regulate and moderate the circulating fluid with effect; but the head is subject to disease, and falls a prey to the baneful consequences of long-continued increased action, or determination of blood, as readily as other parts of the system; and it is reasonable to suppose, if its bony covering could controul its circulation, that a restraint so equable and permanent would defeat the efforts of irregular action.

I am, &c.

*Bedford, Oct. 7, 1805.* JOHN PULLEY.

